# Bone Regeneration Using Adipose-Derived Stem Cells in Injectable Thermo-Gelling Hydrogel Scaffold Containing Platelet-Rich Plasma and Biphasic Calcium Phosphate

**DOI:** 10.3390/ijms19092537

**Published:** 2018-08-27

**Authors:** Han Tsung Liao, Ming-Jin Tsai, Manuri Brahmayya, Jyh-Ping Chen

**Affiliations:** 1 Department of Plastic and Reconstructive Surgery and Craniofacial Research Center, Chang Gung Memorial Hospital, Chang Gung University School of Medicine, Kwei-San, Taoyuan 33305, Taiwan; lia01211@gmail.com (H.T.L.); brahma.orgchem@gmail.com (M.B.); 2 Department of Chemical and Materials Engineering, Chang Gung University, Kwei-San, Taoyuan 33302, Taiwan; duo8704024@hotmail.com; 3 College of Medicine, Chang Gung University, Kwei-San, Taoyuan 33302, Taiwan; 4 Research Center for Food and Cosmetic Safety, Research Center for Chinese Herbal Medicine, College of Human Ecology, Chang Gung University of Science and Technology, Taoyuan 33302, Taiwan; 5 Department of Materials Engineering, Ming Chi University of Technology, Tai-Shan, New Taipei City 24301, Taiwan

**Keywords:** scaffold, hydrogel, thermo-gelling, biphasic calcium phosphate, platelet-rich plasma, adipose-derived stem cells

## Abstract

For bone regeneration, a biocompatible thermo-gelling hydrogel, hyaluronic acid-*g*-chitosan-*g*-poly(N-isopropylacrylamide) (HA-CPN) was used as a three-dimensional organic gel matrix for entrapping rabbit adipose-derived stem cells (rASCs). Biphasic calcium phosphate (BCP) ceramic microparticles were embedded within the gel matrix as a mineralized bone matrix, which was further fortified with platelet-rich plasma (PRP) with osteo-inductive properties. In vitro culture of rASCs in HA-CPN and HA-CPN/PRP/BCP was compared for cell proliferation and osteogenic differentiation. Overall, HA-CPN/PRP/BCP was a better injectable cell carrier for osteogenesis of rASCs with increased cell proliferation rate and alkaline phosphatase activity, enhanced calcium deposition and mineralization of extracellular matrix, and up-regulated expression of genetic markers of osteogenesis. By implanting HA-CPN/PRP/BCP/rASCs constructs in rabbit critical size calvarial bone defects, new bone formation at the defect site was successfully demonstrated from computed tomography, and histological and immunohistochemical analysis. Taken together, by combining PRP and BCP as the osteo-inductive and osteo-conductive factor with HA-CPN, we successfully demonstrated the thermo-gelling composite hydrogel scaffold could promote the osteogenesis of rASCs for bone tissue engineering applications.

## 1. Introduction

Autogenous bone graft and bone substitutes are current approaches for bone defect repair after trauma, tumor ablation or infection [1]. Nevertheless, these approaches all have drawbacks such as an unpredictable absorption rate, donor site morbidity, foreign body reaction, possible infection and unpredictable bone regeneration [2]. For the healing of bone defects, a tissue engineering approach combining stem cells with osteogenic property with a suitable scaffolding material can be suggested. Therefore, bone tissue engineering (BTE) is now a popular research topic for bone defect repair/regeneration, which consists of three main factors, i.e., cells, scaffolds and growth factors [3]. The revelation of such an idea is that stem cells could be osteo-induced into the osteoblast lineage by the action of growth factors that function as signaling molecules within the bone-mimetic three-dimensional (3D) scaffold for bone tissue formation [4].

Bio-ceramics such as calcium phosphates are attractive for BTE applications due to their biocompatibility, bioactivity, degradability and osteo-conductivity [5]. In addition, an added advantage of using calcium phosphates is their structural and chemical similarity to the mineral phase of native bone [6]. The most widely investigated calcium phosphates that are used for bone regeneration and repairs are hydroxyapatite (HAP), β-tricalcium phosphate (β-TCP), and their mixture biphasic calcium phosphate (BCP). As an ideal bone substitute, BCP is the preferred ceramic material, due to its controllable degradability, high bio-resorption rate and favorable biological properties [7]. Indeed, evolution of the concept of using macroporous BCP, which contained HAP and β-TCP mixed in various ratios, for bone regeneration has been reported nearly 30 years ago [8,9]. The usefulness of BCP stemmed from the preferential dissolution of β-TCP compared to HAP, which allowed the manipulation of the biodegradation rate of BCP with different HAP/β-TCP ratios [10]. Although calcium phosphates are known to enhance osteoblast proliferation and differentiation, these ceramic materials are brittle and difficult to mold in order to fill irregular bone defects [11,12]. Besides, the dispersion of calcium phosphate particles during implantation may be difficult in both ectopic and bony sites [13].

Hydrogels are soft materials with 3D cross-linked network formed from hydrophilic homopolymers or copolymers. Other than using their 3D networks for cell entrapment, hydrogels can absorb a large amount of water and allow for diffusion of nutrients and metabolites from the cells. As a scaffolding material for tissue engineering applications, hydrogels could fulfill the requirement to temporarily provide an extracellular matrix (ECM) environment for the cells during the early stage of tissue regeneration [14]. This will be followed by replacement of the hydrogel matrices by a natural ECM secreted by the cells as the tissue formation process proceeds [15].

By responding to environmental stimuli for sol-to-gel transition, such as change in temperature, pH, light or ionic strength, a physical in situ forming hydrogel is one of the most popular injectable scaffolds for BTE [16]. Using the in situ forming hydrogel, an injectable scaffold could be molded to fit a bone defect of irregular shape, shorten the operation time, lessen the post-operative discomfort and fasten post-surgical recovery. In view of the popularity of minimally invasive surgery, cell delivery in such an injectable hydrogel scaffold by endoscope could be suggested as a feasible clinical application of BTE [17]. Being a particular kind of injectable in situ forming hydrogel responsive to temperature change, a thermo-gelling hydrogel shows sol-to-gel phase transition upon heating up to a temperature above its critical transition temperature [18,19]. Poly(N-isopropyl acrylamide) (PNIPAM) is a typical thermo-gelling polymeric hydrogel commercially available in the market. However, non-biodegradability, low biocompatibility and the toxicity of degradation products limit its biomedical applications [20,21]. Therefore, PNIPAM has been modified with many natural polymers, including chitosan, collagen, or hyaluronic acid, to form PNIPAM-based copolymers with better biodegradability, increased cell attachment and improved cell proliferation, without changing the sol-to-gel phase transition characteristics [22,23,24,25]. 

Chitosan is a linear polysaccharide derived from chitin and composed of glucosamine and N-acetyl glucosamine linked by β(1→4) covalent bonds. In recent years, biomaterials based on chitosan have been applied in BTE, which was demonstrated to be osteo-inductive, and promote cell proliferation and mineral-rich matrix deposition when used for osteoblast culture [26]. Interestingly, previous studies have reported that chitosan-modified calcium phosphates could enhance the mechanical strength of the inorganic phase when used as a scaffold for BTE applications [27,28]. Hyaluronic acid (HA) is a non-sulfated glycosaminoglycan formed from repeating disaccharide units of N-acetyl-d-glucosamine and d-glucuronate with β(1→4) and β(1→3) bonds. HA was shown to have strong effects on cell–cell interactions and migration [29]. As a scaffold for BTE, HA-based materials were proved to be suitable for bone regeneration in many studies [30,31]. 

Considering the advantages of those natural biomaterials for BTE, we have successfully prepared a thermo-gelling hydrogel copolymer, hyaluronic acid-*g*-chitosan-*g*-poly(N-isopropylacrylamide) (HA-CPN) and demonstrated its potentials as a BTE scaffold using bone marrow-derived stem cells from in vitro and in vivo ectopic bone formation studies [32]. Follow up studies by incorporation of osteo-conductive BCP microparticles into a HA-CPN scaffold further proved HA-CPN/BCP to be a suitable injectable composite hydrogel scaffold for human fetal osteoblast culture with improved osteoblastic differentiation and ECM mineralization, which formed ectopic bone tissue from nude mice subcutaneous implantation experiments [33]. Nonetheless, osteoblasts are not a good cell source for BTE. Adult stem cells are better sources due to their ability of self-renewal and multi-potent differentiation. Among them, adipose-derived stem cells (ASCs) seemed to be a preferred cell source for clinical application as harvesting adipose tissue is easy and safe using local anesthesia. Furthermore, higher amount of ASCs within fat tissue could be harvested compared to harvesting bone marrow-derived stem cells from the bone marrow aspirate and the harvesting procedure is associated with less donor site morbidity [34].

As stated before, growth factor is also one of the key components for BTE. Therefore, scaffolds for enhanced bone regeneration should be endowed with growth factors retaining and releasing properties to mediate accelerated bone regeneration. Indeed, allografts and xenografts are combined with growth factors as the osteo-inductive substances for natural bone-like performance [35]. Consider that a complete bone healing cascade depends on a wide range of growth factors, it could be suggested that incorporation of a spectrum of necessary growth factors would be a more rational approach compared with solely relying on a specific growth factor for BTE [36]. 

Platelets in the blood play an important role during the early stage of wound healing by taking part in blood clot formation and creating the right microenvironment to guide and control the healing cascade [37]. For more efficient bone repair by external use of platelets, enriching platelets in the plasma by removing red blood cells represents a feasible approach. This enriched blood plasma, platelet-rich plasma (PRP), is a promising alternative approach to enhance bone formation as it is a rich source of growth factors originating from α-granules in PRP, which contain various growth factors, such as platelet-derived growth factor, transforming growth factor-β, epidermal growth factor, insulin growth factor and vascular endothelial growth factor [38,39]. Abundant examples in the literature have addressed the positive effects of PRP on bone regeneration by combining with different biomaterials and various cell sources [40]. Improvement of osteogenesis of ASCs by PRP was also confirmed in a previous study by us [41]. Therefore, we hypothesize the incorporation of PRP and BCP as the osteo-inductive and osteo-conductive factor in the thermo-gelling hydrogel HA-CPN would influence the osteogenesis of ASCs. 

To test this hypothesis, we evaluate the biological response of rabbit adipose-derived stem cells (rASCs) in HA-CPN and HA-CPN/PRP/BCP scaffolds in this study. The injectable scaffolds were first compared for rASCs differentiation into the osteogenic phenotype in vitro, followed by evaluation of HA-CPN/PRP/BCP composite scaffolds seeded with rASCs for regeneration of critical-size rabbit cranial defects in vivo.

## 2. Results and Discussion

### 2.1. Cell Proliferation

As an autogenous blood fraction without transmissible infectious agents, PRP is endowed with preferred properties for clinical use such as a high platelet concentration and being free from hypersensitivity reactions [42]. The platelet concentration in our prepared PRP was determined to contain 3.2 ± 0.4 × 10^9^ platelet/mL, which is ~10 times that in the blood collected for preparing PRP. It should be noted that a platelet concentration higher than 1 × 10^9^ platelet/mL was reported to be suitable for the clinical application of PRP [38].

**Figure 1:**
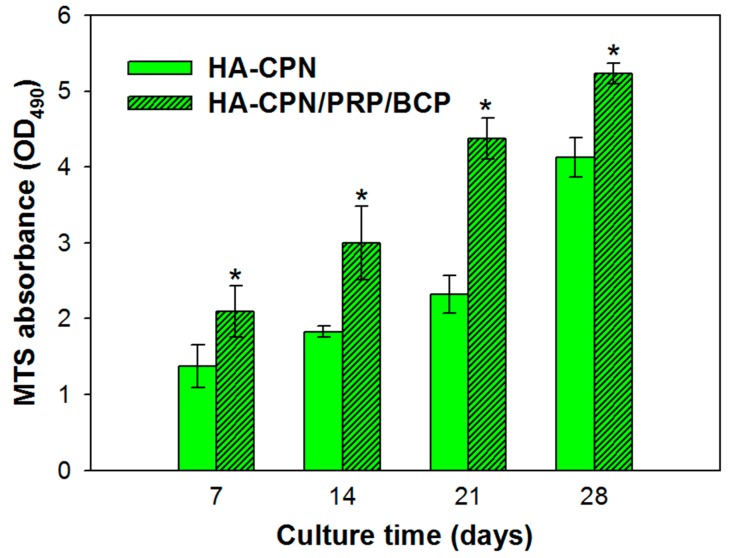
Proliferation of rabbit adipose-derived stem cells (rASCs) in hyaluronic acid-*g*-chitosan-*g*-poly (N-isopropylacrylamide) (HA-CPN) and HA-CPN/platelet-rich plasma (PRP)/biphasic calcium phosphate (BCP) thermo-gelling hydrogel scaffolds. * *p* < 0.05 compared with HA-CPN.

As shown in Figure 1, rASCs proliferated steadily in HA-CPN and HA-CPN/PRP/BCP from day 7 to day 28 as revealed from the continued increase of the optical density (OD_490_) value using the 3-(4,5-dimethylthiazol-2-yl)-5-(3-carboxymethoxyphenyl)-2-(4-sulfophenyl)-2H-tetrazolium (MTS) cell proliferation assay kit, which is directly correlated with the number of viable cells. At the same seeding density, there were more viable cells in HA-CPN/PRP/BCP than in HA-CPN with significant difference found throughout the culture period (*p* < 0.05), which indicates HA-CPN/PRP/BCP provided a better environment for rASCs proliferation than HA-CPN. This result is consistent with a previous report showing HA/gelatin/PRP hydrogel loaded in BCP scaffolds could enhance proliferation rate of MC3T3-E1 pre-osteoblast cells [43]. We also reported that incorporation of BCP into HA-CPN could significantly raise the cell proliferation rate of human fetal osteoblastic cells due to the beneficial effects of BCP [44]. Furthermore, enhancement of proliferation rate of ASCs by PRP was reported by us [45] and confirmed by other groups [46,47]. The use of rASCs in HA-CPN/PRP/BCP is expected to play a crucial role in the treatment of bone defect as recent studies on BTE clearly demonstrated the strong commitment of ASCs towards the osteogenic phenotype under appropriate induction conditions [48].

### 2.2. Live/Dead Staining

**Figure 2:**
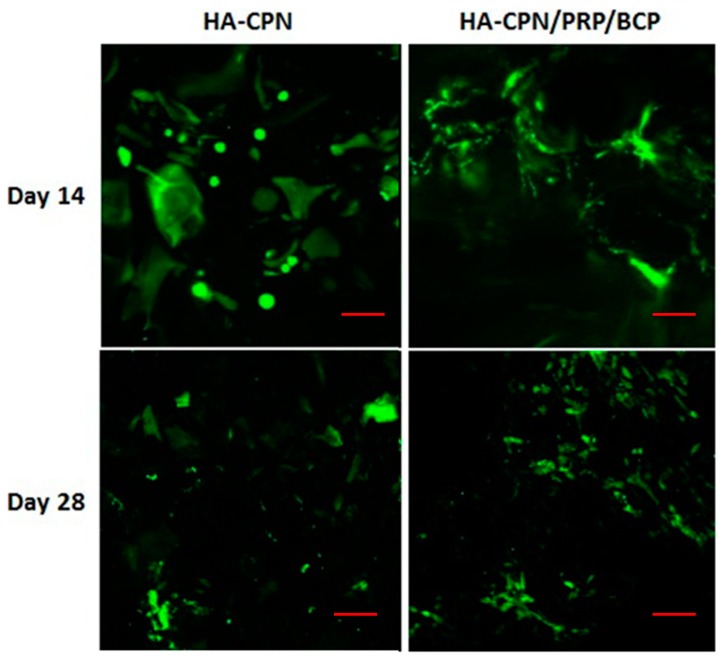
The viability of rASCs in HA-CPN and HA-CPN/PRP/BCP thermo-gelling hydrogel scaffolds by Live/Dead cell viability assays. Bar = 100 μm.

After using MTS assays to confirm the increase of viable cell number in different hydrogels, the Live/Dead fluorescence cell staining assay was used to qualitatively confirm the viability of rASCs with green color representing live cells and red color identifying any possible dead cells. As shown in Figure 2, the Live/Dead staining result revealed high viability of rASCs in both HA-CPN and HA-CPN/PRP/BCP with negligible dead cells found from the confocal image, which underlines the biocompatibility of all hydrogel scaffolds. The number of viable cells (green fluorescence) also gradually increased from day 14 to day 28 for both groups. Nonetheless, more cells were found in HA-CPN/PRP/BCP than in HA-CPN, which is consistent with the MTS results shown in Figure 1. In addition, rASCs started to show a cuboidal morphology of osteoblasts in HA-CPN/PRP/BCP on day 14, indicating accelerated differentiation of cells toward the osteogenic lineage [49]. Taken together, the biocompatibility of HA-CPN/PRP/BCP towards rASCs could be confirmed. 

### 2.3. Alkaline Phosphatase (ALP) Activities

**Figure 3:**
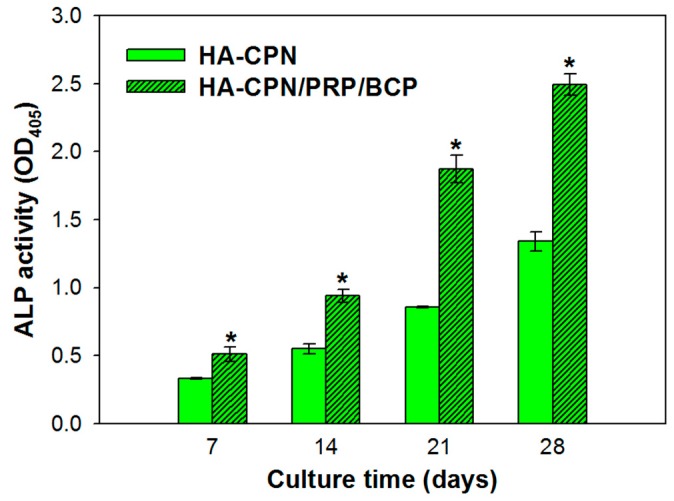
The time-dependent changes of alkaline phosphatase (ALP) activities of rASCs in HA-CPN and HA-CPN/PRP/BCP thermo-gelling hydrogel scaffolds. * *p* < 0.05 compared with HA-CPN.

Initiation of bone mineralization could be noticed by recognizing the alkaline phosphatase (ALP) marker, which as a marker for early osteoblastic differentiation and commitment of stem cells towards the osteoblast phenotype [50]. As nucleation starts with the deposition of calcium with inorganic phosphates and leads to local calcification, hydrolysis of phosphate esters leads to elevated mineralization of ECM and stimulates osteogenic differentiation [51]. Thus, ALP could be considered as a worthy measurement tool to determine the extent of osteo-differentiation of stem cells. Figure 3 indicates continued ALP production throughout the 28-day culture period for both groups; nonetheless, the ALP activity of rASCs in HA-CPN/PRP/BCP was significantly higher than in HA-CPN at all time points. Therefore, combinatory effects from PRP and BCP accelerated rASCs differentiation toward the osteoblast lineage, with ASCs in HA-CPN/PRP/BCP exhibiting enhanced ALP activity. Comparing the trend of ALP expression at various time points in different thermo-gelling hydrogel scaffolds, HA-CPN/PRP/BCP clearly showed dominance in rASCs differentiation [52].

### 2.4. Von Kossa, Alizarin Red and ALP Stains

**Figure 4:**
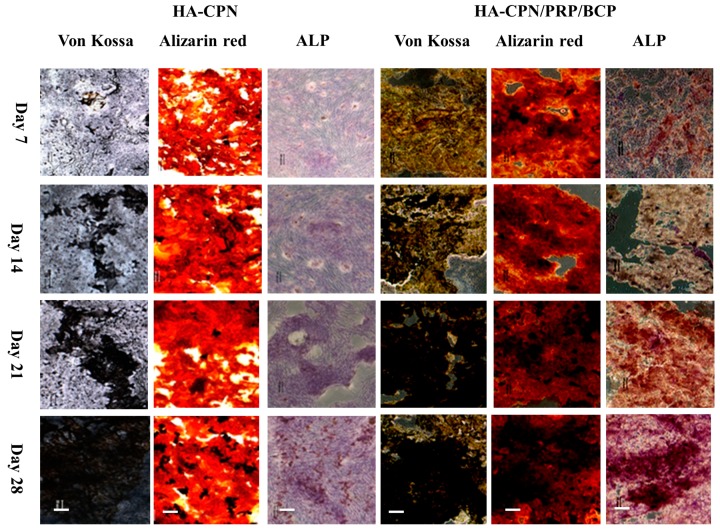
The von Kossa, Alizarin red and alkaline phosphatase (ALP) staining of cryosection slices of rASCs in HA-CPN and HA-CPN/PRP/BCP thermo-gelling hydrogel scaffolds. Bar = 100 μm.

For qualitative evaluation of the mineralization of ASCs in HA-CPN and HA-CPN/PRP/BCP, the cryosection slices of cell/scaffold constructs were subject to von Kossa, Alizarin red (AR) and ALP stains. The mineralized nodules were revealed by staining with von Kossa staining reagents while calcium deposition on matured ECM was determined by AR stain. As shown in Figure 4, both von Kossa and Alizarin red stains revealed time-dependent mineralization of rASCs in HA-CPN hydrogel matrix. Nonetheless, the staining intensity was dramatically enhanced in HA-CPN/PRP/BCP. The concentration of mineralized ECM stained dark brown to black from von Kossa stain increased with culture time and appeared early for rASCs in HA-CPN/PRP/BCP than in HA-CPN, indicating more mineralized nodules formation with rapid development of osteoblast phenotype to form a mineralized matrix, which could be ascribed to the osteogenic natures of incorporated PRP/BCP in HA-CPN. More prominent AR staining was also evident in HA-CPN/PRP/BCP, in which nodules stained in red color originating from calcium ions in mineralized ECM secreted by osteo-differentiated rASCs increased with culture time. Since the capacity to deposit minerals is a marker for mature osteoblasts, it could be concluded that rASCs encapsulated in HA-CPN/PRP/BCP develop into an osteoblast phenotype faster than in HA-CPN, with accelerated mineralization stage to deposit mineralized ECM [53]. 

Considering ALP staining results in Figure 4, positive ALP stains (violet color) for rASCs in HA-CPN/PRP/BCP evidence the increase of intracellular ALP activity with culture time, particularly for cell constructs cultured longer than 2 weeks. On the other hand, minimum ALP staining intensity was observed for HA-CPN. HA-CPN showed light violet color while thick violet color dots with brownish red shadows were observed in HA-CPN/PRP/BCP on day 7. For rASCs in HA-CPN/PRP/BCP, violet and reddish brown violet ALP stains were observed on day 14 and day 21, respectively. The staining intensity turned much stronger and was a dark purple color on day 28, indicating continued elevation of ALP activity in HA-CPN/PRP/BCP. Taken together, the enhanced ALP staining intensity for rASCs in HA-CPN/PRP/BCP strongly endorse elevated ALP activity of rASCs in the presence of PRP and BCP, especially for cell constructs cultured longer than 14 days, which is consistent with the trend observed by biochemical assays (Figure 3).

### 2.5. Calcium Content

**Figure 5:**
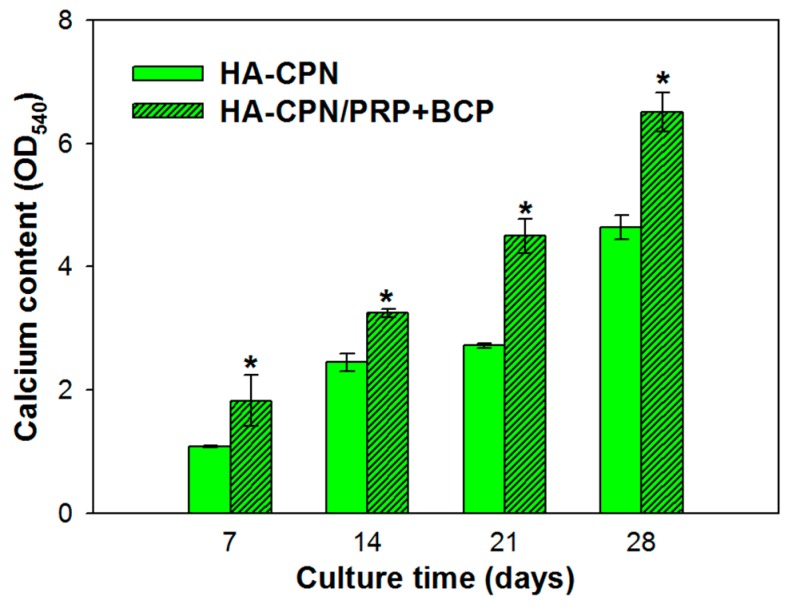
The calcium content of rASCs cultured in HA-CPN and HA-CPN/PRP/BCP thermo-gelling hydrogel scaffolds. * *p* < 0.05 compared with HA-CPN.

A quantitative determination of calcium content was used to cross confirm the calcium-based mineralization of rASCs (Figure 5). By extracting Alizarin red S (ARS) that binds to Ca^2+^ on cell surface with 10% cetylpyridinium chloride, quantitative evaluation of rASCs mineralization could be achieved by determining OD_540_ and augment the qualitative AR staining results shown in Figure 4. Since stem cells start depositing calcium during their later cell proliferation phase, the extent of calcium deposition depends strongly on the duration of the cell culture period [54]. As shown in Figure 5, the calcium content represented by OD_540_ increased rapidly with time from day 7 to day 28 in both hydrogel scaffolds. Nonetheless, the HA-CPN/PRP/BCP group showed significantly higher calcium content than the HA-CPN group throughout the 28-day culture period, which is in agreement with the qualitative AR staining images shown in Figure 4 and in line with ALP activity increase shown in Figure 3. 

### 2.6. Scanning Electron Microscopy/Energy Dispersive X-Ray (SEM/EDX) Analysis

**Figure 6:**
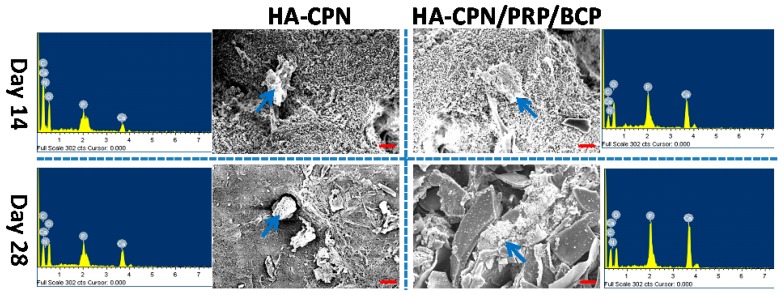
Scanning electron microscopy (SEM) micrographs and energy dispersive X-ray (EDX) measurements for detecting mineralization of rASCs in HA-CPN and HA-CPN/PRP/BCP thermo-gelling hydrogel scaffolds. The arrow in each SEM image indicates the place subject to EDX analysis with the spectrum shown besides the SEM image. Bar = 20 μm.

To examine cell morphology and mineralization, scanning electron microscopy/energy dispersive X-ray (SEM/EDX) analysis of cell/scaffold constructs at day 14 and 28 was attempted and the results are reported in Figure 6. Although cell morphology variance may arise due to rASCs being entrapped in the hydrogel matrix rather than adhered to a substrate surface, a morphological change could still be observed when rASCs were cultured in HA-CPN/PRP/BCP, which showed better cell spreading than in HA-CPN. This implies better osteogenesis of rASCs since stem cells will flatten and spread when undergoing osteogenesis [55]. In addition, the presence of mineralized nodules on the cell surface is higher for rASCs in HA-CPN/PRP/BCP compared to HA-CPN, which underlines the importance of the combinatory cues from PRP and BCP in HA-CPN/PRP/BCP for effective mineralization of rASCs [52]. It is well recognized that mineral formation during stem cell culture implied cellular osteoblastic differentiation [56]. As deposition of calcium phosphate leads to mineralization, the mineralization of inorganic phosphates on cell surface could be deemed as prime evidence for osteo-differentiation of rASCs [57]. Thus, the white mineral deposition from SEM observation could qualitatively identify mineralization, which could be further analyzed quantitatively through elemental analysis by EDX analysis. The EDX spectra associated with each SEM image was shown in Figure 6, from which the atomic percentages of elements in minerals deposited by rASCs were determined for quantitative comparison. As shown in Figure 6, the extent of mineralization increased from day 14 to day 28 and higher percentages of Ca and P were found for HA-CPN/PRP/BCP than HA-CPN. Specifically, the Ca/P atomic percentage was 6.64%/4.52% for HA-CPN/PRP/BCP at day 14 and 7.46%/5.55% at day 28. In comparison, the Ca/P atomic percentage was only 1.01%/0.72% at day 14 and 3.85%/2.74% at day 28 for HA-CPN. Comparing HA-CPN/PRP/BCP with HA-CPN, the Ca and P atomic percentage increase was 6.6-fold and 6.3-fold on day 14 and 1.9-fold and 2.0-fold on day 28, indicating early and accelerated mineralization of rASCs in HA-CPN/PRP/BCP. Furthermore, the Ca/P ratios in HA-CPN and HA-CPN/PRP/BCP were between 1.4 and 1.5 and close to that of bone, whose mineral primarily consists of Ca and P with a Ca/P ratio from 1.4–1.7 [58]. Therefore, the atomic percentage values of Ca and P from EDX thus further demonstrate the potential application of HA-CPN/PRP/BCP for bone regeneration.

### 2.7. Gene Expression by Quantitative Real-Time Polymerase Chain Reaction (qRT-PCR)

**Figure 7:**
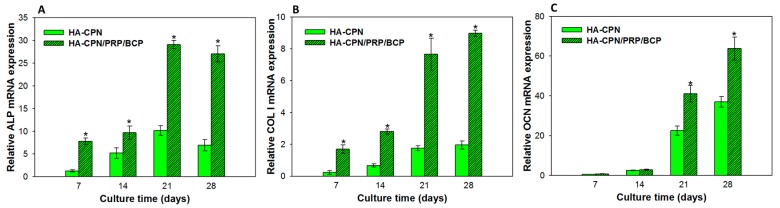
Osteogenic gene expression levels of rASCs after cultured in HA-CPN or HA-CPN/PRP/BCP thermo-gelling hydrogel scaffolds. Alkaline phosphatase (ALP) (**A**), collagen type 1 (COL I) (**B**) and osteocalcin (OCN) (**C**) gene expression was quantified using quantitative real-time PCR.* *p* < 0.05 compared with HA-CPN.

The analysis of osteogenic gene expression could endorse the successful differentiation of rASCs into the osteoblast lineage. Thus, we further compared the relative mRNA expressions of major osteogenic differentiation marker genes (ALP, collagen type 1 (COL I), and osteocalcin (OCN)) of rASCs cultured in HA-CPN and HA-CPN/PRP/BCP (Figure 7). Being expressed at the early to middle stages of osteo-differentiation, ALP and COL were up-regulated during the first 2 weeks of cell culture without further increase of mRNA expression after day 21 [59]. On the other hand, the late expression of OCN could be confirmed from the continued increase of mRNA expression up to 28 days [60]. The scaffold-dependent analysis on gene expression further endorses the effect of PRP/BCP in controlling the expression of various osteogenic markers. Indeed, HA-CPN/PRP/BCP showed a significant enhancement of ALP and COL I gene expression over HA-CPN starting from day 7, which is consistent with their time-dependent gene expression sequence during osteogenic differentiation. On the other hand, being a late marker for osteogenesis, OCN would be up-regulated at a later stage of cell culture. Therefore, a significant difference of OCN mRNA expression was only noted between HA-CPN and HA-CPN/PRP/BCP after 14 days. Overall, we can infer that response of rASCs to BCP as well as PRP in HA-CPN leads to the difference in gene expression levels and control the time sequence of up-regulation of osteogenic genes. Considering the accelerated development of osteogenic phenotype of rASCs in HA-CPN/PRP/BCP in vitro, we proceeded with in vivo experiments to test the in vivo bone regeneration potential of HA-CPN/PRP/BCP/rASCs. 

### 2.8. In Vivo Studies

**Figure 8:**
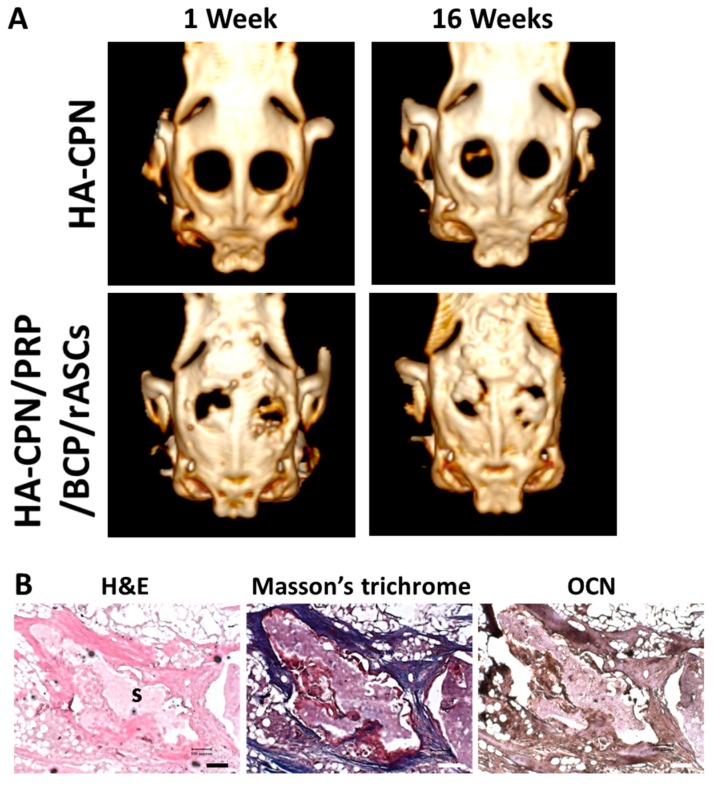
(**A**) The computed tomography (CT) scanning images of rabbit calvarial defects filled with HA/CPN and HA-CPN/PRP/BCP/rASCs 1 and 16 weeks post-implantation. (**B**) Hematoxylin and eosin (H&E), Masson’s trichrome and immunohistochemical staining of OCN of implanted HA-CPN/PRP/BCP/rASCs in rabbit calvarial defects 16 weeks post-implantation. Bar = 100 μm. S indicates remaining scaffold material.

After investigating in vitro osteogenesis, the critical size cranial bone defect model created in rabbits was used to test the in vivo bone regeneration potential of allogenic rASCs in HA-CPN/PRP/BCP. The HA-CPN/PRP/BCP/rASCs construct was cultured in vitro for 21 days prior to implantation. Since the 16-week animal experiment period did not lead to infection or inflammation symptoms around the implant sites and all rabbits stayed alive, we could confirm the in vivo biocompatibility of HA-CPN/PRP/BCP. One and 16 weeks post-implantation, the animals underwent computed tomography (CT) examination to evaluate new bone formation (Figure 8A). The 3D reconstructed CT images at week 1 showed some bone formation starting from one end and penetrated towards the center of the defect zone, without bridging the defect. After 16 weeks, the image density was elevated obviously with bone formation throughout the scaffold. The new bone was compact and bridged between the two ends of the circular defect and the defect was mostly covered with neo-bone formation based on the CT image density. For the control implanted with HA-CPN, negligible bone formation was found from CT images after 16 weeks. After sacrificing all animals at week 16, the specimen was harvested and bone formation was examined through histological evaluation, including hematoxylin and eosin (H&E), Masson’s trichrome and immunohistochemical (IHC) staining of OCN, to cross-confirm the high-density area found in the CT image, was bone tissue (Figure 8B). From the H&E stain, osteoblasts in the bone matrix were observed together with osteoid formation, indicating bone growth in the defect region. Residual scaffold materials (labelled as S) could be observed 16 weeks after in vivo implantation. Masson’s trichrome stain further clearly revealed bone formation as collagen in the osteoid was stained blue in sections of implanted HA-CPN/PRP/BCP/ASCs constructs as differentiated from rASCs deposited bone matrix (osteoid) and were embedded in the newly synthesized ECM. Osteoid is the organic portion of the bone matrix that is secreted by osteoblasts during new bone formation [61]. It contained several specific proteins with COL I being the predominant one and the rest being ground substance. Therefore, the development of new bone tissue may be verified from mineralized osteoid and adjacent bone cells. From IHC staining, the major ground substance in osteoid, OCN, could be identified in tissue sections, confirming that differentiated rASCs displayed an osteoblast phenotype. Taken together, the histological and IHC staining results support active osteogenesis from implanted rASCs and the observation that injected cell mass in the HA-CPN/PRP/BCP scaffold has formed new bone tissue.

## 3. Materials and Methods

### 3.1. Materials

N-isopropylacrylamide (NIPAM) and 2,2-azoisobutyronitrile were acquired from Sigma-Aldrich (St Louis, MO, USA) and were recrystallized prior to use from methanol and hexane, respectively. Chitosan (molecular weight = 1.5 × 10^5^ Da) and 2-morpholinoethane sulfonic acid (MES) was purchased from Sigma-Aldrich (St Louis, MO, USA). N-Hydroxysuccinimide (NHS) and 1-ethyl-3-(3-dimethylaminopropyl) carbodiimide (EDC) were obtained from Acros Organics, Thermo Fisher Scientific (Geel, Belgium). Hyaluronic acid (HA) (molecular weight = 1.3 × 10^6^ Da) was obtained from Bloomage Freda Biopharm Co. (Jinan, China). Dulbecco’s modified Eagle medium (DMEM, Gibco, Thermo Fisher Scientific, Waltham, MA, USA) and fetal bovine serums (FBS, HyClone, Thermo Fisher Scientific, Waltham, MA, USA) were prearranged for cell culture. Biphasic calcium phosphate (BCP) microparticles (0.5–1 mm particle size) contained 60% (*w*/*w*) of beta-tricalcium phosphate (β-TCP) and 40% (*w*/*w*) hydroxyapatite (HAP) were provided by Berkeley Advanced Biomaterials, Inc. (San Leandro, CA, USA). CellTiter AQueous One Solution Reagent for cell proliferation assay was supplied by Promega Life Sciences (Madison, WI, USA). 

### 3.2. Synthesis of Hyaluronic Acid-g-Chitosan-G-Poly(N-Isopropylacrylamide) (HA-CPN)

The HA-CPN copolymer was prepared as reported previously [23]. Briefly, carboxylic acid terminated PNIPAM (PNIPAM-COOH) was readily prepared in benzene by free radical polymerization between NIPAM with mercaptoacetic acid as a chain-terminating agent and 2,2-azoisobutyronitrile as an initiator. Chitosan-*g*-poly(N-isopropylacrylamide) (CPN) was prepared from 0.5 g of chitosan and 5 g of PNIPAM-COOH in 50 mL MES buffer (pH 5.0) using EDC and NHS as crosslinking agents. After reacting for 12 h at 25 °C and 180 rpm, temperature of the solution was raised to 50 °C for complete recovery of the copolymer by thermal precipitation. For the synthesis of HA-CPN copolymer, CPN prepared above was mixed with HA (0.25 g) in 100 mL of 0.1 M MES buffer (pH 5) and the grafting reaction was carried out at 25 °C and 180 rpm for 12 h in the presence of EDC and NHS. After completing the reaction, impurities and residual HA were removed by the thermal precipitation at 50 °C. The final HA-CPN copolymer used for forming the thermo-gelling hydrogel scaffold was purified by dialysis at 4 °C for 4 days and freeze-dried for storage at room temperature in a desiccator.

### 3.3. Preparation of Platelet-Rich Plasma (PRP) 

The preparation of PRP from rabbits by double centrifugation followed the procedures described in our previous report [45]. The procedures were approved by the Institutional Animal Care and Use Committee of Chang Gung University (IACUC Approval No.: CGU13-063, 30 September 2013). In short, ~30 mL of whole blood was withdrawn from ear vessels of rabbits into a tube that contained a citrate phosphate dextrose solution as an anticoagulant. The whole blood was centrifuged at 400× *g* for 10 min to separate the platelet layer from the plasma and red blood cells. The lower red blood cell layer was discarded while the middle platelet layer and the upper plasma layer were collected and further centrifuged at 800× *g* for 10 min. The PRP was collected as the precipitated platelet layer with part of the plasma layer. The platelet number in whole blood and PRP were determined by a hematology analyzer (CELL-DYN Emerald, Abbott, Abbott Park, IL, USA) to check the quality of PRP obtained by this method.

### 3.4. Isolation of Rabbit Adipose-Derived Stem Cells (rASCs) 

The animal experiments were approved by the Institutional Animal Care and Use Committee of Chang Gung University (IACUC Approval No.: CGU13-140, 18 March 2014) and confirmed to the standards of the Association for Assessment and Accreditation of Laboratory Animal Care. Rabbit adipose-derived stem cells (rASCs) were harvested and isolated according to our former report [51]. Briefly, fat tissue was harvested from a rabbit’s inguinal area and diced into small pieces. The diced fat tissue was washed extensively with phosphate buffered saline (PBS), and digested with 0.05% collagenase in a 37 °C water bath shaker at 165 rpm for 30 min. The enzyme activity was neutralized by adding an equal volume of DMEM/10% FBS, and centrifuged at 250× *g* for 10 min to obtain a high density cell pellet. After removing the supernatant, the cell pellet was re-suspended in 160 mM NH_4_Cl and incubated at room temperature for 10 min to lyse the contaminated red blood cells. The cell pellet was collected again by the same centrifugation step, filtered through a 100 μm nylon mesh to remove cellular debris, placed in culture dishes and incubated overnight with DMEM at 37 °C in a 5% CO_2_ incubator for attachment of rASCs to dish surface. Following incubation, the culture dishes were washed extensively with PBS to remove residual no-adherent red blood cells to obtain rASCs-enriched cell population. 

### 3.5. In Vitro Culture of rASCs in HA-CPN and HA-CPN/Platelet-Rich Plasma/Biphasic Calcium Phosphate (HA-CPN/PRP/BCP)

For the culture of rASCs in the thermo-gelling hydrogel, 10% (*w*/*v*) HA-CPN or 10% (*w*/*v*) HA-CPN/10% (*v*/*v*) PRP/3% (*w*/*v*) BCP solution were prepared in DMEM at 4 °C. Suspension of rASCs was mixed at room temperature with 0.4 mL of HA-CPN or HA-CPN/PRP/BCP solution. The mixture was carefully transferred to a 24-well cell culture plate and incubated at 37 °C for 1 h at a cell seeding density of 1 × 10^5^ cells/well. One milliliter of cell culture medium (90% DMEM, 10% FBS, 50 μmol/l L-ascorbic acid phosphate, 100 nmol/L dexamethasone, 10 mmol/L glycerol 2-phosphate and 1% (*v*/*v*) antibiotic–antimycotic) was added to each well and cell culture was carried out at 37 °C in a humidified 5% CO_2_ incubator with medium change on every 2 to 3 days.

### 3.6. Cell Proliferation 

To determine the proliferation of ASCs, the viable cell number was determined by the 3-(4,5-dimethylthiazol-2-yl)-5-(3-carboxymethoxyphenyl)-2-(4-sulfophenyl)-2H tetrazolium (MTS) assay using the CellTiter AQueous One Solution Reagent. After cooling to 4 °C to reverse the cell/hydrogel constructs to a solution state, 200 μL of cold DMEM was added to obtain cell suspension. The MTS assay were performed by adding 20 μL of MTS solution to 200 μL of cell suspension, followed by incubating at room temperature for 3 h in the absence of light. Colorimetric measurement of the formazan product was performed by measuring the optical density value at 490 nm (OD_490_) using an ELISA plate reader (BioTek Synergy HT, Winooski, VT, USA) [24]. 

### 3.7. Live/Dead Assay

The qualitative assessment on the cell viability of rASCs was evaluated using the Live/Dead Viability/Cytotoxicity kit (Molecular Probes, Thermo Fisher Scientific, Waltham, MA, USA). Once cultured for 21 days, the medium was removed from each well and samples were washed three times with PBS. A Live/Dead staining solution contained 2 μM calcein AM (excitation 494 nm, emission 517 nm) and 5 μM ethidium homodimer-1 (EthD-1) (excitation 528 nm, emission 617 nm) was prepared in DMEM, with calcein AM detecting live cell and EthD-1 for dead cell identification. The samples were incubated with the staining solution at 37 °C for 30 min and stained cells were imaged under a Zeiss LSM 510 Meta confocal laser scanning microscope (Carl Zeiss Microscopy GmbH, Jena, Germany). 

### 3.8. ALP Activity

To analyze the intracellular ALP activity, 800 μL cold cell lysis solution containing 0.1% Triton X-100 and 5 mM MgCl_2_ was added to each well after PBS washing. After incubating at 4 °C for 10 min to lysis cells, the solution was centrifuged at 13,000× *g* for 10 min and the supernatant was examined for ALP activity using *p*-nitrophenyl phosphate as a substrate for the enzymatic hydrolysis reaction. Briefly, 50 μL supernatant was reacted with 50 μL *p*-nitrophenyl phosphate (5 mM) for 60 min at 37 °C before adding 50 μL 0.2 N NaOH as a stop solution to denature ALP. The ALP activity was determined from the optical density value of the solution at 405 nm (OD_405_) using an enzyme-linked immunosorbent assay (ELISA) reader. 

### 3.9. Alizarin Red, Von Kossa and ALP Stains 

After detaching the cell-loaded hydrogel from each well, the sample was embedded in an optimal cutting temperature (OCT) compound. The sample in the cryomold was first placed at −20 °C until the OCT compound becomes translucent, followed by transferring to −80 °C overnight. After removing from the cryomold, the frozen block was mounted on a chuck in a cryostat and slices (5 to 10 μm) were collected. The samples were fixed at 37 °C in 4% formaldehyde for 3 h and washed with PBS before subject to different stains. For ALP stain, the sample was stained with ALP substrate solution containing fast blue RR (2 mg/mL) and 1-naphthyl phosphate sodium salt (2 mg/mL) in distilled de-ionized (DDI) water for 30 min at 37 °C. To detect mineralized nodules, Alizarin red stain was used by incubating samples in 2% Alizarin red S (ARS) solution in DDI water for 1 h at 37 °C. For von Kossa stain, samples were pre-treated with silver nitrate solution (5% in DDI water) for 20 min before placed in a UV box for UV light exposure for 2 h. The sample was finally soaked in sodium thiosulfate (5%) for 5 min to remove excess silver nitrate. 

### 3.10. Calcium Content

Alizarin red S (ARS) was used to quantify the calcium ion (Ca^2+^) content in mineralized ECM of osteo-differentiated rASCs. The cell-seeded hydrogel scaffold was removed from wells of the culture plate and washed 3 times with PBS at 37 °C. After fixing with 4% glutaraldehyde solution (in 0.01 M phosphate buffer) for 3 h at 37 °C, 1 mL of ARS solution (2 g ARS in 100 mL DDI water) was added and incubated for 1 h at room temperature. After several washes with DDI water to remove excess dye, the sample was incubated with 1 mL of 10% cetylpyridinium chloride solution to elute the ARS-Ca^2+^ chelating complex. The solution was transferred to an ELISA reader (Synergy HT, BioTek, Winooski, Vermont, VT, USA) and the solution absorbance was determined at 540 nm (OD_540_). 

### 3.11. Scanning Electron Microscopy/Energy Dispersive X-Ray (SEM/EDX) Analysis

The samples were firstly rinsed with 37 °C PBS and then fixed in 4% glutaraldehyde (in 0.1 M phosphate buffer) at 37 °C. After subsequently fixation for another 3 h, the samples were washed 3 times in PBS buffer at 37 °C for 10 min each and then post-fixed in 1% OsO4 (in 0.1 M phosphate buffer) at 37 °C for 2.5 h. Later, samples were washed 3 times with DDI water and dehydrated in stepwise increasing concentrations of ethanol from 50% to 100%. Lastly, the specimens were dried in a critical point dryer, sputter coated with gold, and examined under an S-3000N scanning electron microscope (Hitachi, Tokyo, Japan). The extent of cell mineralization was determined form the atomic percentage of elements using an energy dispersive X-ray (EDX) micro-analyzer (Horiba EX-250, Tokyo, Japan). 

### 3.12. Expression of Osteogenic Genes by qRT-PCR

The expression of osteogenic differentiation marker genes was studied using qRT-PCR following standard protocols for RNA isolation and cDNA preparation. Total RNA of each specimen was isolated with TRIzol reagent (Invitrogen, Carlsbad, CA, USA) and dissolved in RNase-free water. The amount of RNA was determined by measuring the OD value at 260 nm (OD_260_) with a NanoDrop microvolume spectrophotometer (Thermo Fisher Scientific, Waltham, MA, USA). RNA quality was verified by measurement of OD_260_/OD_280_. The cDNA was prepared from 2 μg of total RNA with Revert Aid First Strand cDNA Synthesis Kit in a final volume of 20 μL. The relative mRNA expression of ALP, OCN and COL I osteogenic marker genes was used for study of osteogenesis by designing the primer sequence using the Oligo 6.0 Primer Analysis Software Molecular Biology Insights, Inc. (Colorado Springs, CO, USA). For a single PCR reaction amounting to 20 μL, 0.2 μL of cDNA was used. To make the visualization of PCR products possible in real time, a SYBR Green I Supermix (Yeastern Biotech Co., Taipei, Taiwan) was used. A three temperature cycling, consisting of a denaturation step at 95 °C for 30 s, a annealing step at 57.6 °C for 30 s and an extension step at 72 °C for 30 s, was carried out in an iCycler iQ5 real-time detection system (Bio-Rad Laboratories Inc., Hercules, CA, USA). The specificity of each PCR reaction was assessed by performing melting curve analysis after each reaction. Glyceraldehyde-3-phosphate dehydrogenase (GAPDH) acted as a housekeeping control. Results were quantified for osteogenic marker genes using the 2^−ΔΔ*Ct*^ relative quantification method. The expression of each gene was evaluated in triplicate. 

### 3.13. In Vivo Experiment

The critical size calvarial bone defect model in rabbits was used to evaluate bone formation by implanting HA-CPN/PRP/BCP/ASCs in rabbit skull. Animal protocols were approved by the Institutional Animal Care and Use Committee of Chang Gung University (IACUC Approval No.: CGU13-035). New Zealand white male rabbits weighing 3–4 kg were utilized for the experimental studies. Animals were anesthetized with xylazine (7 mg/kg) and ketamine (140 mg/kg) and the scalp was sterilized with 75% alcohol solution. Animals were kept in single rooms and fed with standard animal feeds. For an assessment of bone regeneration, two 10 mm diameter circular defects were made at the calvarial bone of the rabbit. Five hundred microliter of HA-CPN/PRP/BCP solution containing 5 × 10^6^ rASCs was cultured in vitro for 3 weeks before injecting into each defect. The control group was injected with 500 μL of HA-CPN. Bone formation was examined by computed tomography (CT) examination using a CT scanner (Somatom Sensation 16, Siemens Healthcare GmbH, Erlangen, Germany) 16 weeks post-operation. The CT image acquisition, processing, and manipulation procedures followed standard protocols at a medical facility. The animals were sacrificed 16-week post-implantation with overdosed pentobarbital to retrieve the implants for histological analysis. The samples were fixed in 10% formaldehyde and dehydrated by embedding in paraffin. After sectioning into 5 μm slice sections, hematoxylin and eosin (H & E), Masson’s trichrome and IHC staining of OCN were carried out following standard protocols [62]. The images were recorded under an inverted optical microscope (Olympus IX-71, Tokyo, Japan).

### 3.14. Statistical Analysis

All data are reported as mean ± standard deviation. Tukey’s post hoc test was used to determine the difference between two groups with *p* < 0.05 being considered statistically significant.

## 4. Conclusions

Thermo-gelling HA-CPN/PRP/BCP hydrogel scaffold is found to be a suitable injectable cell carrier for rASCs. In vitro culture of rASCs in HA-CPN/PRP/BCP exhibited better cell proliferation and enhanced osteogenic differentiation. From gene expression, tissue section staining, SEM/EDX and calcium content analysis, PRP/BCP boosts osteoblastic differentiation and ECM mineralization of rASCs in a HA-CPN gel matrix. Using allogenic rASCs in composite HA-CPN/PRP/BCP scaffold to repair critical size calvarial defects in rabbits, the CT analysis confirmed successful bone formation using the cell/hydrogel construct. The phenotypic markers for osteogenesis in the neo-bone tissue could be further identified from histology and IHC staining. Overall, the HA-CPN hydrogel scaffold fortified with PRP/BCP as osteo-inductive/osteo-conductive factors was shown to provide synergistic cues to promote the osteogenesis of rASCs in vitro and in vivo. This composite biomaterial could potentially be useful for development of an injectable cell carrier to assist bone regeneration in a non-load bearing defect site. 

## Abbreviations

HA	Hyaluronic acid
PNIPAM	Poly(n-isopropylacrylamide)
HA-CPN	Hyaluronic acid-*g*-chitosan-*g*-PNIPAM
CPN	Chitosan-*g*-poly(n-isopropylacrylamide)
LCST	Lower critical solution temperature
PBS	Phosphate buffered saline
HAP	Hydroxyapatite
FBS	Fetal bovine serum
DMEM	Dulbecco’s modified Eagle medium
ASCs	Adipose-derived stem cells
rASCs	Rabbit adipose-derived stem cells
ECM	Extracellular matrix
H&E	Hematoxylin and eosin
PRP	Platelet-rich plasma
SEM	Scanning electron microscopy
BCP	Biphasic calcium phosphate
β-TCP	β-Tricalcium phosphate
BTE	Bone tissue engineering
AR	Alizarin red
ARS	Alizarin red S
3D	Three-dimensional
IHC	Immunohistochemical
CT	Computed tomography
OD	Optical density
SEM/EDX	Scanning electron microscopy/energy dispersive X-ray
ALP	Alkaline phosphatase
NHS	n-Hydroxysuccinimide
EDC	1-Ethyl-3-(3-dimethylaminopropyl) carbodiimide
DDI	Distilled de-ionized
MES	2-Morpholinoethane sulfonic acid
OCT	Optimal cutting temperature
qRT-PCR	Quantitative real-time polymerase chain reaction
